# A Study on the Impact of Low-Carbon Technology Application in Agriculture on the Returns of Large-Scale Farmers

**DOI:** 10.3390/ijerph191610177

**Published:** 2022-08-17

**Authors:** Bingbing Huang, Hui Kong, Jinhong Yu, Xiaoyou Zhang

**Affiliations:** 1 Renmin Business School, Renmin University of China, Beijing 100872, China; 2 School of Mechanical Engineering, Beijing Institute of Technology, Beijing 100081, China; 3 School of Economics and Management, Jiangxi Agricultural University, Nanchang 330045, China

**Keywords:** agricultural low-carbon technologies, large-scale farmers, returns, Jiangxi Province

## Abstract

The relationship and mechanism between agricultural low-carbon technology application and farm household returns are not yet clear, especially the lack of evidence from developing countries. This paper takes large-scale farming households in Jiangxi Province, China, from 2019 to 2020 as the research object, and obtains relevant data from field research to explore the intrinsic impact of agricultural low-carbon technology application on the returns of large-scale farming households. Based on the relevant theoretical analysis, the division dimensions of agricultural low-carbon technologies were proposed, and agricultural low-carbon technologies were subdivided into ten specific low-carbon technologies according to six dimensions: tillage system, breeding, fertilization, irrigation, medicine application, and waste treatment. Relevant questions were designed and researched to obtain data on the application status of low-carbon technologies in agriculture and the income cost status of large-scale farmers. Based on the theoretical analysis, the research hypotheses were proposed, and an empirical analysis was conducted based on the obtained data from large-scale farmers. The application of seven low-carbon technologies in agriculture: conservation tillage system, direct sowing technology, selection of compound fertilizer/organic fertilizer/controlled-release fertilizer, soil formula fertilization technology, deep fertilization/irrigation fertilization, sprinkler/drip irrigation/wet irrigation/intermittent irrigation, and straw resourceization significantly improved the income level of large-scale farmers. Furthermore, the application of biodegradable agricultural membranes, biopesticides, and new pesticide-controlled release technologies did not have significant effects on the income level of large-scale farmers, due to their low application and penetration rate. Based on the findings of the paper, the government should strengthen the promotion and subsidies of agricultural low-carbon technologies, especially those technologies that have no significant impact on large-scale farmers’ income, such as biodegradable agricultural membranes, biopesticides, and new pesticide controlled-release technologies, so as to achieve a win-win situation of reducing carbon emissions and increasing farmers’ income.

## 1. Introduction

The history and current status of economic development show that economic development is a function of the dual-factor of technological progress and input factor addition [1]. Compared with other input factors, the decisive role of technological progress in economic development has gradually become more prominent. Francis Bacon, New Atlantis, Marx, and others recognized the important role of technological progress in economic development earlier. Technological progress is the basic driving force of economic development, while the level of socio-economic development and demand conditions determine the direction of technological progress and provide the fundamental driving force for it [2]. Agriculture is an important part of the economy, so the development of agriculture also depends on technological progress [3]. Under the background of “carbon peak and carbon neutralization”, it is not enough to rely only on the progress of traditional agricultural technology, but also pay more attention to the development of agricultural low-carbon technology [4]. With a large population, scattered agricultural resources, and scarce agricultural resources per capital, China must adopt new agricultural technologies to break the constraints of agricultural production resources in order to achieve sustainable agricultural development [5]. Agricultural low-carbon technologies, as a collection of low-consumption, low-pollution, low-emission, and high-efficiency agricultural production technologies, are of great significance in promoting the transformation of China from traditional to modern agriculture [6]. The sustainable development of Chinese agriculture requires both improved economic efficiency and reduced environmental pollution, which means that the chemically high-carbon agriculture of the past cannot continue to be used, and this provides the fundamental impetus for the progress and promotion of low-carbon technologies in agriculture [7]. At the same time, the progress and application of low-carbon technologies in agriculture will certainly have a huge driving effect on rural economic development [8]. The mutual driving role of agricultural low-carbon technologies and rural economic development provides a basic analytical framework for the study of agricultural low-carbon technologies and the drivers of returns for large-scale farmers [9].

The existing literature focuses on the carbon reduction effect and driving factors of specific agricultural low-carbon technologies, and pays less attention to the economic consequences of the application of agricultural low-carbon technologies, which limits the popularization and application of agricultural low-carbon technologies to a certain extent [10,11,12,13]. In order to overcome this problem, this paper is committed to studying the impact of the application of agricultural low-carbon technology on the income of large-scale farmers, and enriching the research on the economic consequences of the application of agricultural low-carbon technology. In order to verify the actual impact of the application of agricultural low-carbon technology on the income of large-scale farmers, the sample data of large-scale farmers in Jiangxi Province, China is used.

This paper has the following three contributions relative to the previous literature: (1) the existing literature on low carbon technologies in agriculture mainly focuses on the drivers of low carbon technologies in agriculture, but less on the economic consequences of low carbon technologies in agriculture. This paper explores the impact of agricultural low-carbon technologies on the returns of large-scale farmers and provides theoretical support for the long-term sustainable diffusion of agricultural low-carbon technologies. (2) The existing literature on low-carbon technologies in agriculture focuses on specific technologies, such as drip irrigation and soil testing and fertilization, and there is less literature on low-carbon technologies in agriculture in a holistic manner. This paper constructs an agricultural low-carbon technology index based on the characteristics and application of agricultural low-carbon technologies as a standard to measure the degree of application of agricultural low-carbon technologies. The construction of the agricultural low-carbon technology index can help solve the problem that agricultural low-carbon technologies are complex and difficult to measure. (3) This paper adds evidence with Chinese characteristics to the study of agricultural low-carbon technologies by taking the field surveyed large-scale farmers in Jiangxi Province, China, as the research object. Jiangxi Province is a representative agricultural province in central China. Jiangxi has a high forest coverage and a relatively low level of economic development, which represents the situation of major underdeveloped regions in China. Therefore, it is highly representative to carry out agricultural low-carbon technology research in Jiangxi Province. Conducting a study on the impact of its agricultural low-carbon technology application on returns is conducive to revealing bottlenecks in the diffusion of agricultural low-carbon technologies in developing countries, as well as promoting the application of agricultural low-carbon technologies.

Based on the discussion and analysis of existing literature, this paper puts forward the research hypothesis of agricultural low-carbon technology driving the income of large-scale farmers. Combined with the survey data of large-scale farmers in Jiangxi Province, China, empirical research and draws corresponding conclusions and policy suggestions are carried out. This paper is structured as follows: Part II is a literature review and the formulation of research hypotheses; Part III is a dimensional classification of low-carbon technologies in agriculture; Part IV is an empirical study, including descriptive statistics of the sample and research hypotheses.

## 2. Literature Review and Research Hypothesis

### 2.1. Literature Review

Numerous scholars have worked on specific agricultural low-carbon technologies and their impacts. Agricultural low-carbon technologies are a collection of technologies that can reduce agricultural carbon emissions in the production stage of agricultural products (cradle-to-farm gate), such as biochar technology, the in-use of agricultural waste, and cropping systems [14,15,16]. Biochar technology, as an emerging low-carbon technology in agriculture, can improve soil fertility, reduce agricultural carbon emissions, and is economically feasible [17,18]. The reuse of agricultural waste is beneficial in reducing carbon emissions [12]. No-till can play a key role in reducing greenhouse gas emissions and simultaneously increasing soil carbon sequestration, thus contributing to climate change mitigation [19]. The application of low carbon technologies in agriculture is key to decoupling agricultural economic growth from CO_2_ emissions [20]. Low carbon technologies in agriculture have the potential not only to mitigate soil fertility degradation but also to increase the agricultural productivity of farmers [21]. Agricultural low-carbon technologies complement conservation agriculture to achieve negative emissions from crop production [10]. Meanwhile, advanced crop soil nutrient management, groundwater conservation measures, water-saving irrigation technologies, and low-carbon energy technologies facilitate the reduction of carbon emissions without reducing food production [22,23]. Therefore, agricultural low-carbon technologies are important for reducing carbon emissions and achieving agricultural transformation and upgrading as well as carbon peaking and carbon neutrality [24,25].

Some other scholars have conducted in-depth studies on the drivers of low-carbon technologies in agriculture. Farm household characteristics, such as size, age, and gender, have important effects on low-carbon technology adoption [11]. For example, there are significant differences in the choice of different low-carbon technologies in agriculture among large-scale farmers, medium-level part-time farmers, and low-level (usually smaller) part-time farmers [26,27,28,29]. Larger-scale farmers were more likely to embrace capital-intensive low-carbon technologies in agriculture, such as new varieties, straw recycling, soil testing, and recipe fertilization. Part-time farmers at the medium level were more inclined to accept capital-intensive, labor-saving, or low-risk agricultural low-carbon farming technologies. In contrast, low-level part-time farmers tend to embrace labor-intensive technologies to reduce capital constraints and agricultural risks [13]. In addition to this, a study from Nigeria showed that age, gender, access to credit, education, extension access, farm size, and social group membership were important drivers influencing the adoption of low carbon technologies in agriculture, with drip irrigation being the most preferred low carbon technology in agriculture by Nigerian farmers [30]. Government subsidies also have a significant impact on the adoption of low-carbon technologies in agriculture. Reasonable government subsidies to farmers are conducive to increasing the adoption of low-carbon technologies in agriculture [31]. The adoption of low-carbon technologies in agriculture is also influenced by the level of formal education, and the results of many empirical studies show that the level of education of farmers is positively correlated with technology adoption. Investment in education significantly reduces the proportion of farmers applying unbalanced nitrogen fertilizers and has a significant effect on whether farmers adopt soil and water conservation technologies [23,32]. In addition, factors such as social media participation, information networks and market incentives, credit, and financial constraints also have a significant and positive impact on the intensity of adoption of low carbon technologies in agriculture [33,34,35].

Increased energy consumption due to economic growth is an important cause of carbon emissions, and thus carbon reduction targets may severely reduce agricultural output [36,37,38]. Although the acceptance of low carbon technologies in agriculture is high among many farmers in China, the overall adoption rate is low [39,40]. This is largely due to the fact that the relationship and mechanism between agricultural low-carbon technologies and farmers’ returns are not yet clear, and little literature has focused on this issue.

### 2.2. Research Hypothesis

According to the theory of technological progress, innovation and application of technology are the core drivers of economic development. Agricultural low-carbon technologies are different from traditional high-carbon technologies, which can not only reduce the level of agricultural carbon emissions, but also increase the output and quality of agricultural products. From the perspective of specific agricultural low-carbon technologies, conservation tillage systems, soil testing and formula fertilization, sprinkler irrigation and drip irrigation and other agricultural low-carbon technologies not only reduce the cost of fertilizer and irrigation water for farmers, but also help to improve the yield and quality of crops. At the same time, ecological and low-carbon agricultural products are more likely to be welcomed by consumers, which helps to improve farmers’ income. The economic benefits generated by the organic combination of various production factors need to be based on a certain scale, that is, economies of scale. Therefore, when studying the economic benefits of agricultural low-carbon technology, we investigate and use farmers with a scale of more than 100 mu, so as to improve the credibility of the research. Therefore, the basic hypothesis of this paper is that the application of agricultural low-carbon technologies has a positive impact on the returns of large-scale farmers, which is analyzed as follows.

Tillage systems include no-till, minimum-till, and conventional tillage, among which no-till and minimum-till are conservation tillage systems. The conservation tillage system has significant economic and ecological benefits compared with the traditional conventional tillage system, which can achieve high quality, high efficiency, and high yield in agricultural production [41]. Firstly, in terms of production cost, the conservation tillage system costs significantly less human and material resources than traditional conventional tillage, and it can achieve one-time sowing in the sowing process, avoiding the need to invest in machinery and manpower to turn the land on a large scale, which reduces the production cost. Secondly, from the viewpoint of soil moisture, the conservation tillage system reduces the chance of deep soil contact with air, which effectively maintains soil moisture and reduces irrigation costs. Again, the conservation tillage system increased the nitrogen content in the soil, which was conducive to reducing nitrogen fertilizer inputs and increasing yields. Based on the above analysis, Hypothesis 1 is proposed.

**Hypothesis** **1.** *The conservation tillage system can reduce production costs and help to improve the returns of large-scale farmers.*

Direct sowing and transplanting are the two main methods of rice cultivation in China at present. Direct sowing refers to the direct sowing of dry seeds or seeds that have been soaked and germinated; transplanting includes the basic processes of germination, seedling raising, and transplanting. As a new rice planting technology in recent years, direct sowing saves labor and energy compared with traditional transplanting, and the yield is not much different from that of transplanting, which can reduce the cost and obtain better economic benefits. Based on the above analysis, Hypothesis 2 is proposed.

**Hypothesis** **2.** *The application of direct sowing technology can reduce costs and promote the income of large-scale farmers.*

The agricultural membranes are one of the important carbon sources in the agricultural production process, and crops are often covered with agricultural membranes at the beginning of planting for drought and frost resistance. As a new low-carbon technology in agriculture, the use of biodegradable agricultural membranes can effectively reduce the carbon emissions of agricultural membranes. However, the cost of biodegradable agricultural membranes is relatively high compared to traditional agricultural membranes, and the difference in efficacy between traditional agricultural membranes and biodegradable agricultural membranes is not significant, which increases the cost and is not conducive to economic efficiency. Therefore, the promotion and application of biodegradable agricultural membranes should rely on the government to provide corresponding subsidies to make the use cost of large-scale farmers lower than that of traditional agricultural membranes, in order to effectively improve the utilization degree and realize low-carbon agriculture. Based on the above analysis, Hypothesis 3 is proposed.

**Hypothesis** **3.** *The application of biodegradable agricultural membranes will increase the cost and reduce the income level of large-scale farmers.*

The choice of fertilizer type can have a large impact on crop production. Reducing the application of nitrogen fertilizers and increasing the application of organic fertilizers and compound and controlled-release fertilizers can effectively reduce the greenhouse gas emissions of chemical fertilizers. Studies have shown that the application of compound fertilizers and controlled release fertilizers has a longer and more balanced nutritional effect than the application of nitrogen alone, and can increase yields. In addition, when determining the amount of fertilizer to be applied, the use of soil testing and fertilizer application technology can effectively reduce the waste of ineffective fertilizers, reduce costs and reduce the source of fertilizer carbon. In terms of fertilizer application method, deep application and irrigation fertilization can deliver effective fertilizer nutrients to the lower layer of soil and crop roots, and improve the utilization rate of fertilizer. Based on the above analysis, Hypotheses 4 to 6 are proposed.

**Hypothesis** **4.** *The application of compound fertilizer, controlled-release fertilizer, and organic fertilizer can enhance the profit level of large-scale farmers.*

**Hypothesis** **5.** *The adoption of soil testing and fertilizer application technology can reduce costs and increase the returns of large-scale farmers.*

**Hypothesis** **6.** *Deep fertilization and watering fertilization can improve the utilization rate of chemical fertilizers and enhance the profit level of large-scale farmers.*

Irrigation methods include conventional irrigation as well as the new sprinkler, drip, wet irrigation, and intermittent irrigation. Sprinkler irrigation is the use of decentralized nozzles for rotary water spraying irrigation; drip irrigation is the irrigation method through fine pipes to irrigation water to the roots of the crop; wet irrigation is to make the field near saturation of water, but does not establish a water layer of irrigation; intermittent irrigation is a certain interval of time, using intermittent water supply to the furrow or border in successive water supply, the use of wave surge flow in the furrow border to advance to wet the soil Irrigation method. Too much water in the soil may lead to problems such as low ground temperature, which may affect the normal growth and development of crops. Compared with the traditional large water irrigation the above four irrigation methods can achieve significant water saving, reduce irrigation water, improve irrigation efficiency, maximize the crop growth water demand and increase yield. In addition, the above four irrigation methods consume less power than traditional pumping flood irrigation, thus reducing carbon emissions. Based on the above analysis, Hypothesis 7 is proposed.

**Hypothesis** **7.** *The scale farmers using sprinklers, drip, wet irrigation as well as intermittent irrigation can achieve better economic benefits.*

The use of pesticides is another important source of carbon in agricultural production. Excessive pesticide application not only increases carbon emissions and agricultural production costs, but also threatens food safety and causes environmental pollution. Biopesticide is a general term for a series of new modern pesticides, mainly including biomass pesticides and biochemical pesticides. Compared with traditional pesticides, biopesticides are derived from animals, plants, or microorganisms and can repel pests without increasing the burden on crops, improving the quality and yield of agricultural products. The adoption of new pesticide-controlled release technologies can slow down the volatile process of pesticides, increase efficacy, reduce the use of pesticides, and lower the cost of agricultural production. Based on the above analysis, Hypothesis 8 and Hypothesis 9 are proposed.

**Hypothesis** **8.** *The adoption of biopesticides can enhance the level of returns of large-scale farmers.*

**Hypothesis** **9.** *The adoption of new pesticide-controlled release technologies can reduce the cost of agricultural production and increase the level of returns of large-scale farmers.*

The treatment of agricultural waste is a key aspect of low-carbon technology application in agriculture, the most important of which is the fertilizer/feed utilization of straw. Returning straw resourceization can improve soil fertility, maintain soil temperature, inhibit water evaporation and increase crop yields. Burning straw not only leads to air pollution and a large increase in carbon emissions, but also reduces soil fertility and is not conducive to increasing crop yields. Based on the above analysis, Hypothesis 10 is proposed.

**Hypothesis** **10.** *Straw composting/overstocking can improve soil fertility, increase yields, and raise the level of returns for large-scale farmers.*

## 3. Study Design

### 3.1. Data

After the questionnaire design, questionnaire surveys were conducted successively: first, the subject group members went to Fuzhou city and Ganzhou city from 15 July to 15 August 2019, and with the assistance of the agricultural bureaus of Fuzhou city and Ganzhou city, they selected key townships in the counties where large-scale farmers were concentrated to conduct field interviews in the countryside; second, with the help of Jiangxi Province’s Second, with the help of the “One Village, One Product” college student project carried out in Jiangxi Province, members of the research team selected large-scale farmers who came to study at the College of Continuing Education of Jiangxi Agricultural University to conduct interviews from 20 December to 25 December 2020; Third, they went to the Jiangxi Provincial General Survey Team and Jiangxi Provincial Bureau of Statistics to access the information needed for the study. The total number of questionnaires obtained was 350, of which 322 were valid, as shown in Table 1.

As can be seen from Table 1, the effective sample is mainly concentrated in Ganzhou and Fuzhou regions, accounting for more than 80%, mainly because Ganzhou and Fuzhou regions are relatively concentrated in the distribution of large-scale farmers in Jiangxi province, which is also the focus area of our preliminary research.

### 3.2. Methodology

In this paper, the dependent variable is set as profit (*y*); the independent variables are the 10 agricultural low-carbon technology variables set above; and five additional control variables are set as population($x_{11}$), age ($x_{12}$), education level ($x_{13}$), party cadre ($x_{14}$), and planting scale ($x_{15}$), as shown in Table 2. 

Since the scale variables have different units and take different values, the above variables, except nominal and ordered variables, are uniformly logarithmic when performing regression analysis, as shown in Equation (1).

*lny* = *α*_1_ ∗ *x*_1_ + *α*_2_ ∗ *x*_2_ + *α*_3_ ∗ *x*_3_ + *α*_4_ ∗ *x*_4_ + *α*_5_ ∗ *x*_5_ + *α*_6_ ∗ *x*_6_ + *α*_7_ ∗ *x*_7_ + *α*_8_ ∗ *x*_8_ + *α*_9_ ∗ *x*_9_ + *α*_10_ ∗ *x*_10_ + *α*_11_ ∗ *lnx*_11_ + *α*_12_ ∗ *lnx*_12_ + *α*_13_ ∗ *lnx*_13_ + *α*_14_ ∗ *lnx*_14_ + *α*_15_ ∗ *lnx*_15_ + *θ* (1)

In Equation (1), y represents the net profit per mu for large-scale farmers. $x_{1}$ to $x_{10}$ represent tillage system to straw resourceization ten agricultural low-carbon technologies, respectively, $x_{11}$ to $x_{15}$ represent household size to planting scale in control variables, *α*_1_ to *α*_15_ are variable coefficients, and *θ* denotes residuals.

## 4. Results and Discussion

### 4.1. Descriptive Statistics

#### 4.1.1. Family Characteristics

The average age of household heads in the research area was 47 years old. Only three households were under 30 years old, accounting for 0.9%; 63 households were under 40 years old, accounting for 19.62%; and 87 households were between 40 and 45 years old, accounting for 27.1%. This indicates that the farmworkers of large-scale farming households are generally older, mainly distributed over 40 years old, and these people have greater difficulties in accessing new knowledge and generating new ideas. In addition, 138 of the household heads are party members and cadres, accounting for 43%, a high proportion, indicating that party members and cadres play a pioneering and exemplary role in carrying out large-scale agricultural operations, or it may be that there is a shortage of young and strong rural laborers, and the older generation of laborers who remain in rural areas take up the main administrative work in rural areas. In terms of education level, the education level of heads of large-scale farm households is mainly distributed in high school and below, accounting for 87% of the total, with only 13% share of the university and above, and the largest number of heads of households with junior high school education level. This indicates that the education level of large-scale farm household heads is generally not high, and even university education has many informal full-time graduates, and the overall cultural level is low, which is not conducive to the acceptance and application of new agricultural low-carbon technologies. In terms of household size, the total household size of the studied large-scale farming households is five persons on average, and the labor force is 2.7 persons on average, and the agricultural labor force is 2.2 persons on average. It can be seen that the agricultural labor force is less than half of the total household size, and part of the labor force has been diverted to non-agricultural jobs.

#### 4.1.2. Production Characteristics

**Figure 1:**
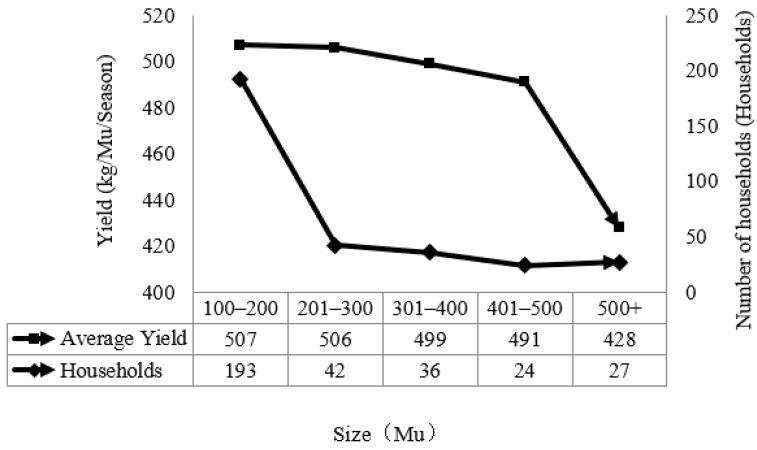
Scale of cultivation and production by large-scale farmers.

An important aspect of the production characteristics is the scale of planting. The object of this paper is the scale of farmers, defined as farmers with a planting area of 100 mu and above, and in the research process we focused on the selection of local large grain growers, as shown in Figure 1.

As can be seen from Figure 1, the average yield of large-scale farmers decreases gradually with the expansion of the planting scale, from 507 kg/mu/season to 428 kg/mu/season. According to the database of the economy prediction system, the total grain output of Jiangxi Province in 2019 was 21,575 million kg, with a sown area of 55 million mu, corresponding to 392 kg per mu. Due to the large planting area, the output per mu of farmers surveyed is significantly higher than the average value. Meanwhile, it can be seen that with the expansion of the planting scale, the labor consumed by farmers on the unit of land is decreasing and the substitution of machinery is strengthening, thus the high yield of intensive farming cannot be maintained. Farmers’ scale is mainly concentrated between 100–200 mu, with 193 households, nearly 60% of the share. This indicates that the scale of production of farmers in the research area is limited, and most of them are still “small-scale operations” on the scale, mainly because there are more hilly and mountainous areas in Jiangxi Province and less concentrated and contiguous arable land.

#### 4.1.3. Cost-of-Revenue Characteristics

**Figure 2:**
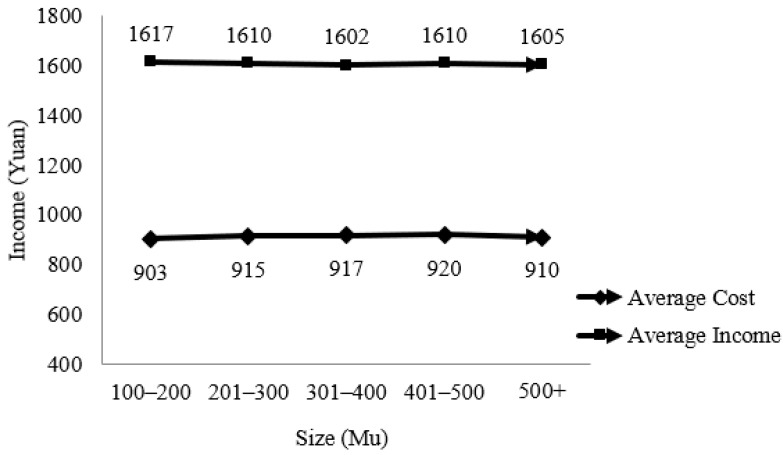
The cost of income for large-scale farming households.

The sample size farmers are mainly large grain farmers, and rice sales are their main source of income. In order to ensure the comparability of data, we only counted the sales income and production cost of rice. The relationship between income cost and land size is shown in Figure 2.

It can be seen from Figure 2 that the income and cost of rice sales remain basically stable at different scales. When the scale is small (100–200 mu), the cost is low; as the scale gradually expands, there is a small increase in production cost; after the scale reaches a certain level (500 mu or more), there is a small decrease again. This may be because with the expansion of the planting area, the degree of machinery instead of labor increases, management costs rise, and has not yet achieved economies of scale; after the scale reaches a certain level, the fixed costs tend to stabilize, and the total variable costs increase with the scale, the unit cost decreases, achieving economies of scale.

#### 4.1.4. Low Carbon Technology Characteristics in Agriculture

Based on the complexity and systematic nature of low carbon technologies in agriculture, identifying and quantifying low carbon technologies in agriculture is the core problem to be addressed in this paper. The academic research on agricultural low-carbon technologies generally follows the traditional technology research paradigm, which simply classifies agricultural low-carbon technologies into binary variables of adoption/non-adoption, on the basis of which the influencing factors of agricultural low-carbon technologies are studied. Pathak and Aggarwal (2012) systematically studied the cost, classification, and improvement directions of agricultural low-carbon technologies, taking wheat and rice as an example in the Indus-Ganges plain of India, which provides an important reference for China [14]. The 10 agricultural low-carbon technologies classified by Xiong et al. (2021) are fertilizer reduction, organic fertilizer mixed with chemical fertilizer, soil testing, application of controlled-release fertilizer, deep fertilizer application, breeding new varieties, promoting conservation tillage, alternate drainage, water-drought rotation (rice-oilseed rape/rice-wheat), and pesticide reduction [16].

Low-carbon technologies in agriculture are applied throughout the whole process of agricultural production, and they are reflected in different cropping patterns and crops. Therefore, there is no unified academic standard for the classification of low-carbon technologies in agriculture, but some core classifications are mostly the same. In the process of agricultural production, fertilizers and pesticides are used as the main carbon sources, and the technologies for fertilizer and pesticide application are also used as the core agricultural low-carbon technologies, in addition to breeding, irrigation, and transportation. Scholars have put forward different classifications of agricultural low-carbon technologies. Although they are referred to in different ways, the basic connotations are similar, mainly around breeding, fertilizer, pesticide, irrigation, waste, cropping systems, and machinery, covering the whole process of agricultural production. Based on the basis and consideration of scholars’ research, this paper divides agricultural low-carbon technologies into the following seven dimensions according to the agricultural production chain, as shown in Table 3.

The quantification of agricultural low carbon technologies is the basis for conducting a study on the drivers of agricultural low carbon technology applications on the returns of large-scale farmers. After the dimensional division and explicit classification of low carbon technologies in agriculture, 10 nominal variables for low carbon technologies in agriculture are set based on the design problem. After obtaining data on the application of low carbon technologies in agriculture, the level of application of agricultural low-carbon technologies is reflected by the mean value of each agricultural low-carbon technology application, as shown in Equation (2).

*A*_*i*_=∑C_*ij*_/*N* (2)

**Figure 3:**
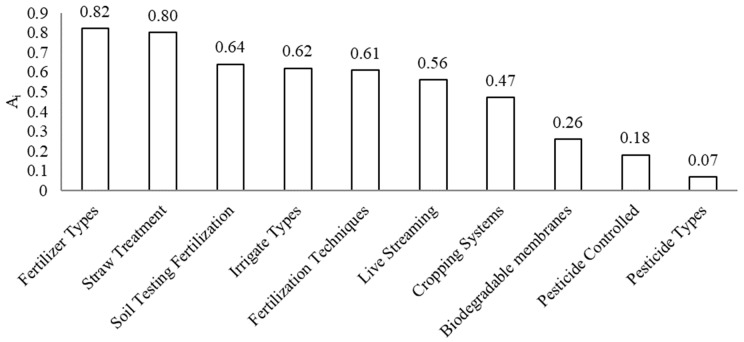
Average status of low-carbon technology applications in agriculture.

In Equation (2), $A_{i}$ represents the average value of the *i*th agricultural low-carbon technology, $C_{ij}$ represents the application status of the *i*-th agricultural low-carbon technology (expressed as 0/1) of the *j*-th scale farm household, and *N* represents the number of sample scale farm households. The mean value of each agricultural low-carbon technology was calculated by this formula, and the specific application levels are shown in Figure 3.

As can be seen in Figure 3, the application level of each agricultural low-carbon technology varies widely. The application levels of straw resourceization and fertilizer types exceed 0.8, with a high prevalence rate; the application levels of pesticide types, as well as new pesticide, controlled release technologies are below 0.2. Biopesticides are still rare in the actual agricultural production process, and the use of new pesticide-controlled release technologies is also low.

### 4.2. Regression Results

The adoption of low-carbon technologies in agriculture not only reduces the level of agricultural carbon emissions, but also improves the efficiency of agricultural operations and increases income. Conservation tillage systems can increase soil moisture retention and nutrients, and improve yields. The application of organic fertilizers, compound fertilizers, and controlled-release fertilizers can improve the utilization efficiency of chemical fertilizers and increase soil fertility; soil testing and fertilizer application can maximize yields according to local conditions; deep fertilizer application and irrigation application can improve the utilization efficiency of fertilizers. New irrigation technologies such as sprinkler and drip irrigation can improve irrigation efficiency and increase yields. New pesticide-controlled release technology and bio-pesticides can control pests and diseases more environmentally friendly and improve the growing environment of crops. Straw resourceization can improve soil fertility, moisturize and increase temperature, and increase yield. Based on the questionnaire data, a linear regression of the model was conducted and the results are shown in Table 4.

As can be seen in Table 5, the model fit is high and the model fit (R-squared) for the effect of low carbon technologies in agriculture on profit (model 3) is 0.743, indicating that 74.3% of the variance in the dependent variable can be revealed by the model and the model is significant, as shown in Table 5.

As can be seen in Table 5, the regression sum of squares accounts for approximately 80% of the total (outlying) sum of squares, indicating that the model explains 80% of the variation in the dependent variable, and F = 21.4, sig = 0.00 < 0.05, indicating that the model is generally significant and has a high degree of confidence.

Table 6 shows the results of the regression analysis, and the variance inflation factor is basically at 1.6 and below, indicating that there is no significant covariance problem. From the regression results, among the independent variables agricultural low carbon technologies, $x_{1}$ (tillage system), $x_{2}$ (direct sowing), $x_{4}$ (fertilizer type), $x_{5}$ (fertilizer application method), $x_{6}$ (soil testing and fertilizer application), $x_{7}$ (irrigation method), and $x_{10}$ (straw resourceization) have a significant positive effect on the income of large-scale farmers, and among the control variables,$\;x_{15}$ (planting scale) has a negative effect on the profit of large-scale farmers.

The survey data from large-scale farmers in Jiangxi Province, China show that the application of agricultural low-carbon technologies such as conservation tillage system, direct sowing technology, the use of controlled-release fertilizer, deep fertilization, soil testing, and formula fertilization, drip irrigation, sprinkler irrigation, and straw resourceization have a significant positive impact on the income of large-scale farmers. (1) Consistent with Afshar and Dekamin (2022), the results show that conservation tillage systems can reduce the cost of agricultural production, improve income, and increase the returns of large-scale farmers [42]. The conservation tillage system reduces the cost of tilling compared with the traditional tillage system, and also improves the nutrient and moisture environment of the soil, increasing crop yields. (2) The adoption of direct sowing technology in breeding sessions can reduce the cost of seeding sessions and increase the yield. Scopel et al. (2005) and Botta et al. (2007) also find that direct sowing technology can increase production while reducing carbon emissions [43,44]. Direct sowing technology reduces the processes of seedling preparation, seed dipping and germination, and seedling sowing compared with traditional transplanting, which reduces the production cost of large-scale farmers and increases the level of income. The adoption of biodegradable agricultural membranes has no significant effect on the yield, probably because the application of biodegradable agricultural membranes is less and more costly in actual production. In addition, the technology of degradable agricultural membranes is still immature, which also limits its large-scale application and cost reduction [45]. (3) The application method of fertilizer, the selected varieties and the basis of application all have a significant impact on the returns of large-scale farmers. The use of compound fertilizer, organic fertilizer, and controlled-release fertilizer can balance soil nutrients, improve soil fertility and increase yield; the adoption of soil testing and formula fertilization technology can improve the efficiency of fertilizer application, reduce the amount of fertilizer applied and improve economic benefits [46,47,48]. (4) The adoption of new irrigation methods such as sprinkler irrigation, drip irrigation, wet irrigation, and intermittent irrigation can improve irrigation efficiency and reduce water costs, while helping to maintain soil temperature, improve soil hydrological environment and increase yield [49,50,51]. (5) The return of straw resourceization has a significant positive impact on the returns of large-scale farmers. Through decomposition and fermentation, straw can improve soil organic matter content, increase soil fertility, and maintain ground temperature, increase crop yield, and raise the income level of large-scale farmers. Furthermore, straw returning to farmlands could counterbalance all of the K_2_O, the majority of the P_2_O_5_, and a portion of the N in chemical fertilizers. Promoting the return of straw to field has a great potential to reduce the use of chemical fertilizer, air pollutant emission, and environmental burden [52].

However, the adoption of new pesticide-controlled release technologies and biopesticides has no significant impact on the returns of large-scale farmers, mainly because the adoption of biopesticides and new pesticide-controlled release technologies in the actual production process is very small and difficult to form a scale. Most large-scale farmers do not know about biopesticides, and the practical application is more difficult. New pesticide-controlled release technology is also relatively small, scale farmers in the use of pesticides generally do not consider the use of controlled release agents, mainly on the function and role of pesticide controlled release agents and cost-effective understanding is not in place. In addition, new pesticide-controlled release technologies and biopesticides are still immature, which also limits their low-cost promotion and application [53,54].

Furthermore, the increase in household size can raise the income level, and the expansion of the planting scale reduces the unit income level. The increase of labor force can enhance the fine management of unit farmland and increase the unit output, while the expansion of planting scale reduces the level of fine cultivation of unit farmland and lowers the unit output. The effects of increasing household size and expanding planting scale on the level of returns of large-scale farmers are logically consistent intrinsically.

## 5. Conclusions

The impact of low-carbon technologies in agriculture on the returns of large-scale farmers is systematically explored in this work. Based on the characteristics and application of low carbon technologies in agriculture, an agricultural low carbon technology index is constructed to measure the application of these technologies. Then the impact of low-carbon technologies on the returns of large-scale farmers in Jiangxi Province, China, was investigated through a field survey.

The application of seven low-carbon technologies in agriculture: conservation tillage system, direct sowing technology, selection of compound fertilizer/organic fertilizer/controlled-release fertilizer, soil formula fertilization technology, deep fertilization/irrigation fertilization, sprinkler/drip irrigation/wet irrigation/intermittent irrigation, and straw resourceization could significantly improve the income level of large-scale farmers. However, the application of biodegradable agricultural membranes, biopesticides, and new pesticide-controlled release technologies did not have significant effects on the income level of large-scale farmers, probably because the application of the above three agricultural low-carbon technologies in the actual agricultural production process was relatively small and the penetration rate was not high, and the effects on income were not significant. 

## 6. Suggestions

Based on the findings of the paper, some suggestions are proposed.

(1) Promote conservation tillage systems. Conservation tillage systems include no-till and minimum tillage, which can enhance soil carbon storage and significantly reduce the carbon emission level of farmland compared to traditional conventional tillage systems, and are key agricultural low-carbon technologies that return farmland from a carbon source to a carbon sink. In addition, a conservation tillage system increases soil organic matter content, regulates soil temperature, enhances soil moisture retention capacity, and improves yield, while reducing tillage costs and increasing economic benefits. Therefore, conservation tillage systems have significant synergistic effects between achieving low carbon agriculture and modernizing agriculture. In the actual production process, most farmers do not have a high awareness of the conservation tillage system, and they are still stuck in the traditional farming mindset that “you get what you put in, you get what you harvest”, and that more tillage is necessary for high yield. To promote the conservation tillage system, on the one hand, we should strengthen the technical training of farmers, through technology in the countryside, centralized lectures, and other ways to make farmers aware of the advantages of the conservation tillage system; on the other hand, through the agricultural cooperatives, family farms and other local agricultural organizations of scale, so that the majority of farmers see the conservation tillage system can generate real income, driving the implementation of the conservation tillage system.
(2) Promote direct sowing technology and encourage the use of biodegradable agricultural membranes. Although there is some decrease in yield compared to the traditional transplanting method, the overall benefit of rice cultivation is significantly increased. In addition, direct sowing technology eliminates the tedious process of traditional seed breeding and reduces the carbon emissions of the breeding process. The adoption rate of direct sowing technology in rice cultivation is gradually expanding, but there is still a significant proportion of large-scale farmers who have not yet used direct sowing technology. The reason for this may be that there are some problems with direct seeded rice technology, such as the increase in weeds, the climatic risk of inversion, and the high mechanization requirements for sowing and harvesting, so the acceptance is not yet high enough. The promotion and application of direct sowing technology should, on the one hand, strengthen the education and training efforts and the application of large households to drive the efforts, and on the other hand, actively solve the problems arising from the application of direct sowing technology, and improve the applicability of direct sowing technology. The popularity of biodegradable agricultural membranes is low mainly because the application of biodegradable agricultural membranes has little effect on improving economic efficiency, and the cost is high compared with traditional agricultural membranes. To promote the application of biodegradable agricultural membranes, it is necessary to strengthen the policy subsidies, improve the tax preferences and subsidies in production and use, reduce the production and use costs of biodegradable agricultural membranes, and make large-scale farmers take the initiative to adopt biodegradable agricultural membranes through government regulation and market-oriented approach.
(3) Improve the science and efficiency of fertilizer application. Fertilizer is the first major source of carbon emissions from agriculture, to reduce agricultural carbon emissions, mainly from the use of chemical fertilizers to think of ways. But the growth of crops cannot be separated from chemical fertilizers, which have a great impact on the production results of crops. China is the largest fertilizer user in the world, and for every ton of fertilizer applied, the agricultural output value will increase by 1,245,910,000 RMB. Therefore, improving the science and efficiency of fertilizer application is the only way to achieve low carbon fertilizer use and high-efficiency income. First of all, in fertilizer selection, the use of compound and controlled release fertilizers, as well as organic fertilizers in the selection of fertilizers reduces the application of traditional nitrogen fertilizer. The use of compound fertilizer and organic fertilizer can balance soil nutrients, improve soil fertility and increase yield, while reducing carbon emissions, and the application of controlled-release fertilizer can prolong fertility and reduce the application of chemical fertilizers. Secondly, in the application method, deep fertilization or watering fertilization is used instead of the traditional spreading of fertilizer. Deep fertilizer application and irrigation fertilization can improve the efficiency of fertilizer utilization and reduce the amount of fertilizer, while increasing the yield. Finally, soil testing and fertilizer application techniques are used to determine the amount and type of fertilizer to be applied. Traditional fertilizer application is based on the amount of fertilizer applied in previous years or the amount of fertilizer applied by other local farmers, ignoring the nutrient demand of the soil. Soil formula fertilization can meet the individual needs of the soil, while reducing the waste of useless chemical fertilizers, lowering the soil burden, increasing yields, and improving economic benefits. In the actual production process, large-scale farmers have low awareness of the scientific nature of fertilizer application, and the lack of science in the amount of fertilizer used, the type of fertilizer applied, and the way of fertilizer application has made chemical fertilizer the number one source of carbon in agriculture, and also reduced economic benefits. To improve the science and efficiency of fertilizer application, we should strengthen the training of farmers on fertilizer application and raise their awareness of environmental protection. At the same time, agricultural extension personnel should also go deep into the farmers, go into the fields to give scientific guidance to large-scale farmers, and lead them to apply fertilizer scientifically to realize the synergy of agricultural economic benefits and environmental benefits.
(4) Promote the use of new irrigation technology. Compared with the traditional large water irrigation, sprinkler irrigation, drip irrigation, moist irrigation, and intermittent irrigation can improve water use efficiency, reduce water use costs and improve economic benefits. In the sample study area, it is not common for large-scale farmers to adopt new irrigation technologies, and almost no farmers use sprinkler and drip irrigation, while some farmers use wet irrigation and intermittent irrigation. The cost of sprinkler and drip irrigation is higher than that of wet irrigation and intermittent irrigation, and the cost of water saved is not enough to compensate for the equipment cost of sprinkler and drip irrigation, thus the popularity rate is low. To promote new irrigation technologies, we should increase policy support and provide greater subsidies for sprinkler and drip irrigation equipment, so that farmers can afford to buy and use them. At the same time, strengthen the irrigation technology training for large-scale farmers, deepen the concept of new irrigation, and gradually reverse the backward irrigation situation in rural areas.
(5) Promote the application of biopesticides and pesticide-controlled release formulations. Pesticide application is one of the important aspects of the agricultural production process and one of the important elements in the transformation of traditional agriculture to modern agriculture. The massive use of modern chemical pesticides on the one hand has greatly reduced crop pests and diseases, ensuring high agricultural yields and providing us with abundant agricultural products; on the other hand, it has also brought about serious environmental pollution, including a large increase in agricultural carbon emissions. As early as 1962, the American scholar Rachel Carson shockingly revealed in Silent Spring the great threat posed by the excessive use of pesticides to the human living environment in modern society. Controlling the excessive application of pesticides can not only reduce the cost of pesticide application for large-scale farmers, but also reduce agricultural pollution and carbon emissions, which is conducive to achieving a deep synergy between the economic and ecological benefits of agriculture. The promotion and application of biopesticides can significantly improve the current situation of pesticide pollution, and the elimination of pests and diseases through biological laws and mechanisms is the future development direction of agriculture. The new pesticide-controlled release technology can extend the efficacy of traditional pesticides, reduce the overuse of pesticides, and reduce the environmental risks brought by pesticides. In the actual agricultural production process, the application of biopesticides is very little, and the adoption of emerging pesticide controlled release technology is also less, mainly because the technology of biopesticides is still in the formative stage, is not mature, the application cost is very high, emerging pesticide controlled release technology also has a high application cost, the scale of farmers on emerging pesticide controlled release technology has not been really recognized. Promote the application of biopesticides can be emerging pesticide control release technology, on the one hand, the drug to strengthen basic research, improve the applicability of biopesticides, and pesticide control release technology to reduce costs; on the other hand, to strengthen the publicity and education of large-scale farmers, so that biopesticides and new pesticide control technology in the vast rural areas to gain recognition; and finally to strengthen policy subsidies to improve the application of large-scale farmers biopesticides and emerging pesticide The enthusiasm of controlled release technologies.
(6) Improve the application rate of straw resourceization. The traditional way to deal with straw is to burn or abandon it regardless, which not only wastes the nutrients of the straw itself, but also greatly increases agricultural carbon emissions. Straw resourceization can improve the soil organic matter content, while regulating soil temperature, enhancing moisture storage capacity, and can increase yields without raising costs, improving economic efficiency. Eighty percent of the survey respondents have returned straw resourceizations, indicating that straw is commonly returned to the fields in the actual agricultural production process. For the application of straw resourceization of large-scale farmers, on the one hand, enhance the technical training of straw resourceization, improve the scientific nature of straw resourceization of large-scale farmers, improve the efficiency of straw utilization, and maximize the utility of returning to the field; on the other hand, further promote the application of straw resourceization, improve the popularity of straw resourceization, and strive to achieve the popularization of straw resourceization.
(7) Improve the research and development innovation of agricultural low-carbon technologies, and provide a number of agricultural low-carbon technologies with low cost and good benefit. The promotion and application of low-carbon technologies in agriculture are not only conducive to enhancing the economic benefits of large-scale farmers, but also can achieve agricultural carbon emission reduction, which is the only way to achieve sustainable development of modernized high-carbon agriculture. To promote the application of low-carbon technologies in agriculture, farmers are the main body, the government is the leading, and technology is the key. The government should play a leading role in promoting the application of low-carbon technologies in agriculture and provide support for the promotion and application of various low-carbon technologies in agriculture. First, promote the research and development innovation of agricultural low-carbon technologies, and provide a number of agricultural low-carbon technologies with low cost and good benefit. Second, promote the construction of agricultural carbon sink market, and at the same time provide measurement support for farmers’ carbon emission reduction, provide a carbon trading platform for large-scale farmers to achieve carbon emission reduction, and enhance the enthusiasm for large-scale farmers’ carbon emission reduction. Third, drive the application of agricultural low-carbon technologies with new agricultural business entities (agricultural cooperatives and family farms). Fourth, strengthen the publicity and education for farmers to raise the low-carbon awareness of large-scale farmers. Fifth, propose a national development plan for low-carbon agriculture to provide legal support for the promotion and application of low-carbon agricultural technologies. Sixth, strengthen the construction of rural financial markets, encourage the development of new agricultural financial organizations, and provide financial support for the promotion and application of low-carbon agricultural technologies.

## Figures and Tables

**Table 1:** Distribution of the sample.

Region	County/District	Sample Size	Subtotal
Fuzhou city	Dongxiang county	27	131
	Linchuan county	34	
	Nanfeng county	14	
	Linchuan county	24	
	Nanfeng county	18	
	Le’an county	14	
Ganzhou city	Ningdu county	19	114
	Shicheng county	18	
	Xingguo county	23	
	Xinfeng county	33	
	Ningdu county	21	
Jiujiang city	De’an county	13	13
Nanchang city	Jinxian county	18	33
	Anyi county	15	
Xinyu city	Yushui district	17	17
Yichun city	Fengxin County	14	14
Aggregate		322	322

**Table 2:** Variable definitions.

Variable Type	Variable Code	Variable Name	Calculation Method
Implicit Variable	y	Margins	Profit per acre
Independent variable	$x_{1}$	Cropping systems	No-till, minimum tillage = 1; conventional tillage = 0
	$x_{2}$	Direct sowing	Adopted = 1; not adopted = 0
	$x_{3}$	Agricultural membranes	Degradable agricultural membranes = 1; non-degradable agricultural membranes = 0
	$x_{4}$	Type of fertilizer	Ammonium phosphate type fertilizer/organic fertilizer/controlled release fertilizer = 1; other fertilizers = 0
	$x_{5}$	Fertilizer application method	Deep fertilization/irrigation fertilization = 1; spreading open fertilizer = 0
	$x_{6}$	Soil testing and fertilization	Use = 1; no use = 0
	$x_{7}$	Irrigation method	Wet, intermittent irrigation/sprinkler, drip = 1; conventional irrigation = 0
	$x_{8}$	Type of pesticide	Biopesticides = 1; traditional pesticides = 0
	$x_{9}$	Pesticide control technology	Use = 1; no use = 0
	$x_{10}$	Straw resourceization	Compost to field/over belly to field = 1; incineration/other = 0
Control variables	$x_{11}$	Family size	Family size
	$x_{12}$	Age of head of household	Age of head of household
	$x_{13}$	Education level of head of household	Elementary and below = 1; middle school = 2; high school = 3; college = 4; graduate and above = 5
	$x_{14}$	Head of household party cadres	Yes = 1; No = 0
	$x_{15}$	Planting scale	Planted area

**Table 3:** Classification of low carbon technology dimensions in agriculture.

Segment (of Annelid Worms)	Low Carbon Technologies in Agriculture
Cropping systems	No-till, low-till
Sow seeds	1. Direct sowing 2. Use of biodegradable agricultural membranes
Apply fertilizer	1. Fertilizer type: ammonium phosphate fertilizer (nitrogen and phosphorus compound fertilizer), organic fertilizer, slow-release fertilizer 2. Fertilization techniques: deep fertilization, irrigation fertilization 3. Fertilization basis: soil testing and fertilization
Irrigate	Moist irrigation, intermittent irrigation, sprinkler irrigation, drip irrigation
Apply medicine	1. Type of pesticide: application of biopesticides 2. Pesticide controlled release technology
Litter	Straw resourceization

**Table 4:** Model fit.

Models	R	R^2^	Adjusted R^2^	Errors in Standard Estimates
1	0.883	0.779	0.743	0.229

**Table 5:** Analysis of Variance Table.

Models	Square	Degrees of Freedom	Mean Square	F	Saliency
Return to	16.872	15	1.125	21.400	0.000
Residual	4.783	91	0.053	/	/
Aggregate	21.655	322	/	/	/

**Table 6:** Regression Analysis.

Models	Non-Standardized Coefficient		Standardization Factor	t	Saliency	Covariance Statistics	
	B	Standard Error	Beta			Allowable	VIF
(Constant)	5.341	0.656	/	8.140	0.000	/	/
$x_{1}$	0.280	0.056	0.311	4.975	0.000	0.622	1.606
$x_{2}$	0.215	0.050	0.237	4.291	0.000	0.794	1.259
$x_{3}$	−0.020	0.051	−0.020	−0.393	0.695	0.962	1.039
$x_{4}$	0.218	0.062	0.185	3.511	0.001	0.873	1.145
$x_{5}$	0.248	0.050	0.269	4.987	0.000	0.835	1.197
$x_{6}$	0.250	0.055	0.266	4.557	0.000	0.713	1.402
$x_{7}$	0.222	0.054	0.240	4.089	0.000	0.704	1.421
$x_{8}$	0.032	0.095	0.018	0.341	0.734	0.889	1.125
$x_{9}$	0.072	0.061	0.061	1.184	0.240	0.916	1.091
$x_{10}$	0.124	0.060	0.110	2.067	0.042	0.864	1.158
$\ln(x_{11}$)	0.005	0.079	0.003	0.057	0.955	0.816	1.225
$\ln(x_{12}$)	0.163	0.151	0.062	1.080	0.283	0.737	1.357
$x_{13}$	−0.022	0.028	−0.046	−0.778	0.439	0.696	1.438
$x_{14}$	−0.047	0.051	−0.052	−0.924	0.358	0.778	1.286
$\ln(x_{15}$)	−0.082	0.040	−0.107	−2.036	0.045	0.875	1.143
